# Publishing in the academy: An arts-based, metaphorical reflection towards self-care

**DOI:** 10.1007/s13384-022-00547-y

**Published:** 2022-06-28

**Authors:** Georgina Barton, Annette Brömdal, Katie Burke, Melissa Fanshawe, Vicki Farwell, Ellen Larsen, Yosheen Pillay

**Affiliations:** The University of Southern Queensland, Brisbane, Australia

**Keywords:** Academia, Publish or perish, Accountability, Reflection, Arts-based research, Metaphor, Self-care

## Abstract

Publishing in the academy is a high-stakes activity often used to measure academic staff progress and inform promotion. Many universities have increased pressure on academics, even at the earliest stages of their careers, to publish in high-ranking journals resulting in increased stress and uncertainty. The authors of this paper are members of a writing group in an Australian regional university, established to support each other towards success in quality research and publishing. Over the 2020–2021 summer semester, six members of the group decided to reflect on their experiences, emotions and outcomes throughout the writing process by participating in four reflective arts-based activities. Theoretical frameworks of reflection and metaphor were used to share findings. Strong evidence of having to grapple with meeting university expectations in tension with personal goals and passions was ever-present. The importance of drawing on both personal resources and significant others to manage these tensions through self-care practices was also evident. Implications resulting from this research include recognising the pressures placed on academics to publish only in specifically ranked journals. Overall, the arts-based reflection was critical in uncovering deeper feelings about the pressures of publishing and supporting higher education employees’ well-being and self-care during the writing process.

## Introduction

Recent national policy changes in Australia have intensified the pressure for academics in many universities to publish in high-ranking journals as they seek to improve their comparative institutional academic status (Coaldrake, 2019). The authors of this paper are members of a writing group established to support participating academics in their area of research and publishing within a School of Education at a regional university in Queensland, Australia. Through regular biweekly meetings, the group aims to work towards publishing one quality sole-authored or collaborative paper each semester (approx. 12 weeks). Across the 2020–2021 summer semester, six members of this group reflected on their writing experiences using an arts-based reflective task every three weeks. These tasks included photo elicitations, poetry, collages and a self-selected artwork, with a short, written reflection accompanying each artefact.

Each arts-based reflective activity was designed for the authors to consider how they experienced the writing process and the feelings associated with the pressure to publish. Arts-based methods provided a means for the group to explore and express their experiences beyond traditional forms of discursive communication, or to express the ‘ineffable’, providing a source for contemplation, discussion and openness to possibilities (Barone & Eisner, 2011; Barton, 2019). This process of reflection was guided by the following research questions:

1. What emotions or metaphors arise when reflecting on the writing process and pressure to publish in the academy?
2. To what extent does an arts-based approach to reflecting on the writing process and pressure to publish assist in the preservation of self?

It is intended that findings from this study support others working in universities to embrace the positive aspects of publishing and writing, and that the strategies shared herein may assist other academics in the practice of self-care and care of others.

## A review of the literature

### Publish or perish

Researching and publishing in the academy is a core part of an academic’s working life, yet many scholars have reported on the pressure and stress related to having to publish in high-quality journals persistently and regularly (Weisshaar, 2017). Continuous institutional rhetoric around publishing can be daunting; so much so, that people give up altogether (Cerci & Dumludag, 2019). Several issues have been identified. First, the Q1 stance of universities has been made even more challenging for academics given that such a push has resulted in unprecedented rejection rates by several Q1 journals in recent times. Second, such concern is highly emphasised for early career researchers (those defined as within the first five years post-doctorate) who may still be consolidating their research/publishing skills or experience career disruption through casual employment or family responsibilities (Bosanquet et al., 2017). Additionally, gender roles impact academic career progression (linked to publication output) and women with family and parenting roles are notably impacted (Weisshaar, 2017); a situation amplified during COVID-19 when many women were largely responsible for schooling and/or looking after their children at home (Ribarovska et al., 2021). Moreover, intensified external measures of success (e.g. Excellence in Research for Australia [ERA]) are particularly problematic in regional universities where casualisation of the workforce is extensive and limited support is available for the establishment of pilot research to ensure success in large external research grants (Aprile et al., 2020). Finally, the push to publish only in high-ranking journals disadvantages lower ranked journals that may provide specific issues for academics to discuss. As a consequence, it is essential that academics engaging in reflection to find effective strategies that enable them to meet expectations, maintain a sense of academic agency and practice self-care.

### Preservation of self

Many researchers have noted the significance of protecting self and making sense of the complex conditions in which academics work (Bryan & Blackman, 2019; O’Dwyer et al., 2018). According to Pincus (2006), self-care is something “one does to improve [the] sense of subjective well-being. How one obtains positive rather than negative life outcomes” (p. 1). Richards et al., (2010) further extrapolated that self-care involves preservation of the physical and spiritual self but also involves support from others. Nicol and Yee (2017) also shared several conceptions of self-care including practices in medical contexts with patients and their rehabilitation, and several researchers have explored the idea that an academic life should be fulfilling and valuable, not just a means to an end (Barton, 2019; Manathunga et al., 2017, 2020).

While many methods of self-care reference exercise and stress management, Nicol and Yee (2017) also mentioned a sociological approach to self-care which emanates “from a place of self-love that also has emotional, psychological, and spiritual dimensions” (p. 134). For this paper, we embrace the notion that self-care is how we might best care and nurture our own well-being as well as how we might best support each other during challenging experiences. For us, innovative and creative approaches to practice self-care were paramount as we acknowledged the limitations of just discussing or writing down our feelings. Hence, we adopted arts-based and reflective research methods to support our self-care practices (Moffatt et al., 2016).

### Reflection and arts-based research methods

In theorising the pressures and processes related to publishing and writing in the academy, we chose a creative and reflective method of investigating the phenomenon. Arts-based research offered us an ideal platform to express our feelings related to publishing. Arts-based research can be defined as an activity or conceptual moment involving aesthetic responses to human experience (Barton et al., 2020). Often involving artistic processes, arts-based research methods accept that meaning can be represented in multiple ways, using resources that move beyond text-based communication, such as visual images, music and dance, poetry, and literature and so on. (Barone & Eisner, 2011). The focus is on the process rather than the product so that the researcher can explore particular phenomenon in context (Sinner et al., 2006). They can be used to disrupt and extend the qualitative research paradigm as they can “unsettle” assumptions about what constitute knowledge as well as research (Leavy, 2009. p. 9). The creative outcomes or ‘art’ can be the expression of the findings, the discussion, the implications and/or the conclusive statements. This is different from arts-informed research which aims to make creative processes more accessible to diverse audiences (Wang, 2014). The purpose of our research was entirely an individualised approach for each participant first and foremost, even though we also shared out results with each other and in this paper.

We acknowledged the need to engage in more than discussing or writing down our experiences in the hope that this type of text and oral-based reflection might ameliorate stress and anxiety. We used multimodal forms of representing information to provide much needed and alternative ways of expressing our own personal and collective understanding and experience (McKay & Barton, 2018). In this sense, arts-based research methods were selected to assist us in and through the reflection process to elicit more subtle, ‘felt’ insights that might promote positive and transformative ways of working and enacting self-care.

## Background to the study

The writing group referred to in this paper is a small but productive group within a School of Education in a regional university in Queensland, Australia. Each member receives an annual workload allocation of 20% research, 10% service with the remainder as teaching hours. It is possible to request extra research if productivity exceeds what is expected at the academic’s particular level. In response to this requirement, the group aims to work on a sole or co-authored paper with the purpose of submitting to a high-quality journal by the end of each semester (approximately 12 weeks in duration). Members arrange biweekly 3-h writing blocks which are attended via Zoom as individual’s schedules permit. These meetings generally commence with a time of sharing progress, challenges, advice and mutual support, followed by an extended period of focussed writing and a brief ‘check in’ during the last 15 min. The group is supported by a member of the Professoriate who has a keen interest in supporting early career researchers (ECRs). Between meetings, the group often communicates via email, sharing the highs and lows of their writing and publishing endeavours, including acceptances and rejections, and offering to peer-review each other’s work. The culture of the group is that of a ‘safe space’ and has been attributed by several members as foundational to their work as researchers and writers.

For this paper, six members of the writing group decided to reflect on their experiences over the 2020–2021 summer semester through arts-based research methods, with a view to elucidating how the pressures to publish were impacting us individually and collectively as a group.

## Materials and methods

During the summer of writing, we each progressively completed four arts-based reflective activities:

1. Week 1: Take a photo of something that represents how you are feeling about your writing/publishing as a metaphor and write a brief reflection about the image;
2. Week 4: Compose a Haiku poem or select a few words/phrases that represent how you are feeling at this point in the journey;
3. Week 8: Make a reflective collage or *recollage* (Barton, 2019) through a mindful process; and
4. Week 12: Create a final artwork of your choice and write a brief reflection that expresses your feelings at the end of the semester in relation to your writing.

**Fig. 1:**
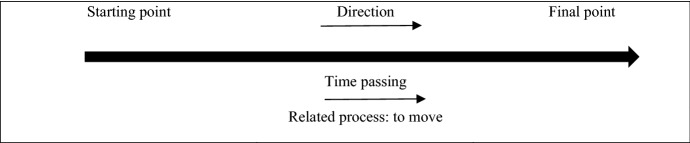
Schema of the metaphor source domain 'path' (Moser, 2000, p. 144)

The framework used to analyse the work was the notion of metaphor. According to Vosniadou and Ortony (1989), metaphors are analogies that can help map personal and social experiences over time. They are also a useful tool to assist in understanding complex topics or new situations (Moser, 2000). Moser stated that “a metaphor consists of the projection of one schema (the source domain of the metaphor) onto another schema (the target domain of the metaphor)” (p. 1). In this way, a sensed metaphor can also relate to a process of growth over time as illustrated in Fig. 1.

Further, Moser (2000) notes that metaphors influence information processing, representing a reliable and accessible operationalisation of tacit knowledge, holistic representations of understanding and knowledge, and social and cultural processes of understanding (pp. 1–2). Therefore, in describing and interpreting our work, metaphor became an important focus as we were reflecting on the challenges and/or successes in our publishing work. It also became evident through the use of metaphors employed in our various artistic responses to the four arts-based activities.

Throughout the project, each member progressively shared their work in a central repository and discussed emerging thoughts and responses. This occurred regularly through shared twice weekly meetings or email which helped to facilitate a sense of ongoing catharsis. At the completion of the four arts-based reflections, the authors met to share the respective impacts of the project and commence a synthesis of their individual reflections over the semester. Given the amount of data, it was decided that each member selected one or two of their artefacts that most holistically expressed their experience, and iteratively re-worked, or “re-presented” (Denzin & Lincoln, 2000) their four accompanying written reflections, thus creating a synthesised culminating written reflection and artistic artefact. Through this process, members were paying particular attention to the use of metaphor in their artefacts, seeking ways that visual and linguistic metaphor were also evident in their written reflections and seeking to draw this out more explicitly in a “lush rendering” through both written and artistic responses that might permit an audience to experience our personal experiences more richly (Barone & Eisner, 2011). These individual re-presentations of the original data from each participant became the findings, which are presented below. The final analysis was enacted through a collective search for, and explication of “threads”: “particular plotlines that threaded or wove over time and place through an individual’s narrative account” (p. 132).

## Findings

Here, we share each authors’ final synthesised arts-based reflection. Each member reflects on their experience through the arts-based process, and how this supported the uncovering of meanings and emotions in relation to publishing in the scholarly field.

### The force of love

**Fig. 2:**
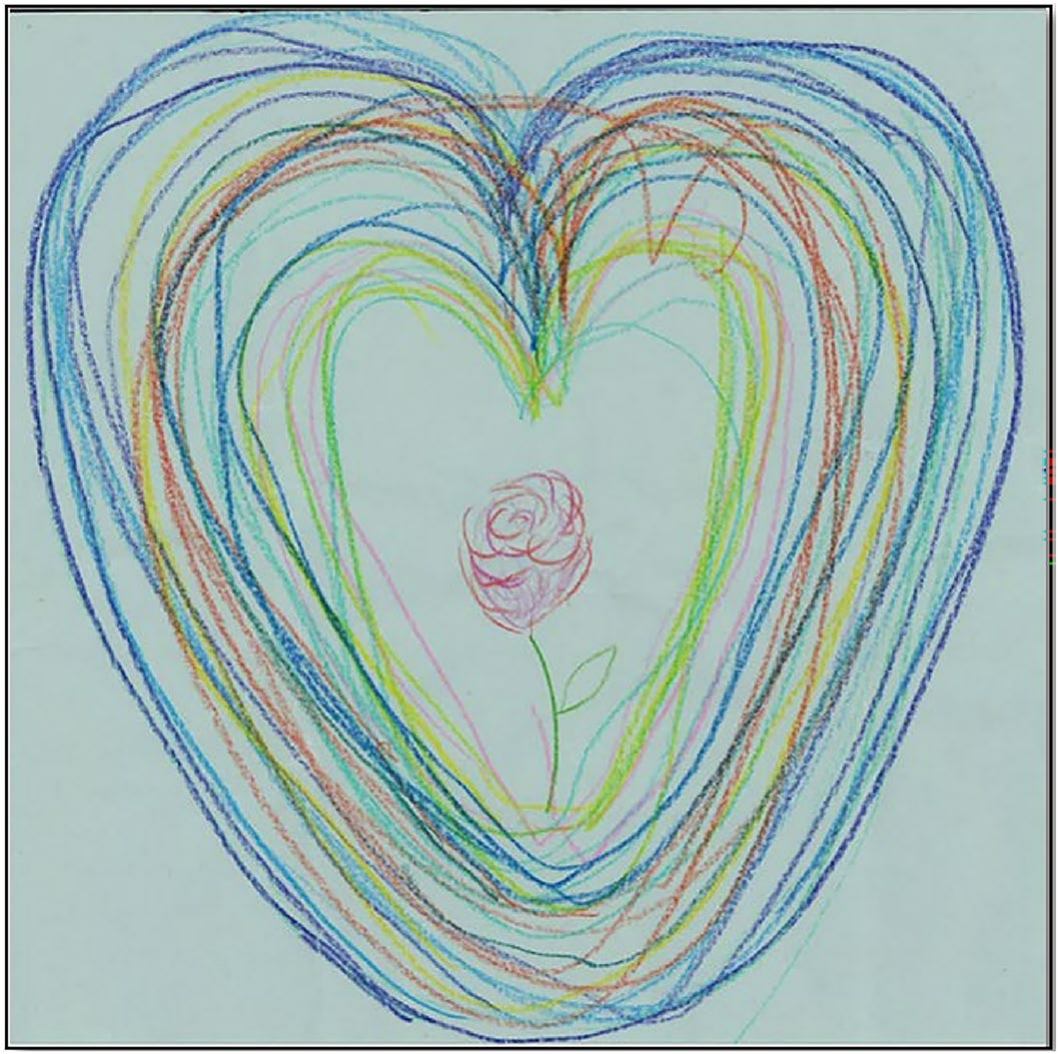
The force of love

I purposefully only engage in research that is based on genuine *love*, to do no harm, and it is strongly rooted in the ethos and philosophy of academic advocacy within the LGBTIQA + space (Fig. 2) (Brömdal et al., 2019, 2020, 2021; Deshman & Hannah-Moffat, 2015). This purpose is often in competition with the ever-stronger neoliberal forces and agenda of universities (Black & Garvis, 2018; Lee, 2014). The pressing research output agenda of universities, and the disconnected workload model I work under, lacking real-time estimations in managing my work (Lindegaard Moensted et al., 2012), could threaten the integrity of my work that starts from a point and *force of love,* depicted by the alternative rainbow love-heart. By no means is this a new ‘non-rosy’ phenomenon, illustrated by the thorny rose in the centre of the love-heart, but it is felt at different times and different junctures in a scholar’s academic journey and career (Coll et al., 2019; Moosa, 2018). Besides loving the research and advocacy work I pursue in the space of gender studies, I love my supportive partner, and being a parent to my little four-year old who is becoming their own strong little person. However, within the current political research and publication climate and landscape, I do feel an immense sense of pressure and stress to balance these three roles and identities satisfactorily without burning out or losing myself in the midst of it (Bueskens & Toffoletti, 2018).

**Fig. 3:**
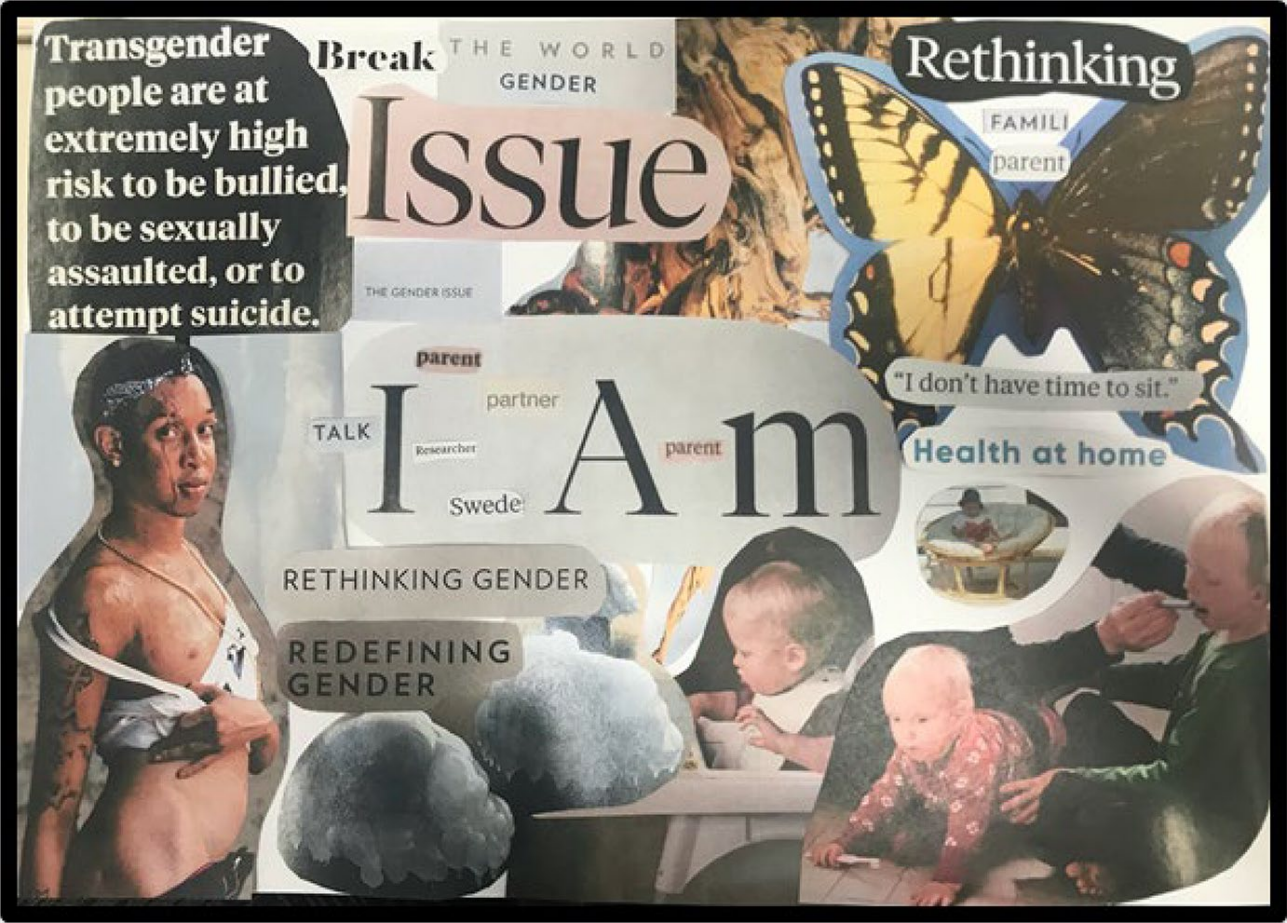
Rethinking who I am

These three competitions for my time and love have forced me to reflect more carefully about how my love for the academic advocacy work I do can come into fruition and bloom without losing the integrity of the research I pursue. Figure 3 above reflects some of these competing forces, feelings and identity-thinking, and my presence in and commitments to them all, while also needing time for ‘me’. I have been devoting more time to reflecting, speed walking or doing Pilates at every lunch break, and blocking out chunks of time to reading and writing every day, purposefully turning off my email/phone (Bryan & Blackman, 2019; Manathunga et al., 2020; Nicol & Yee, 2017), and doing more administrative work in the evenings. Consequently, I am finding some form of peace of mind with how I consciously charge and am charged with love regarding my academic advocacy work. However, to manage my often-unrealistic workload allocated work tasks with my passion for research, I have learned that I must become comfortable with functional work tasks having to wait their turn and be transparent about it. Being true to myself and others about these internal and external struggles, and how I navigate them, nevertheless is the way forward in improving my self-care, finding a sense of balance, and ‘me’.

### The breakthrough

In my research and writing endeavours as an ECR, there is a sense that there must be some ‘divine’ way forward if I could make sense of the complexity, but all too often, this feels out of my reach. While I wrestle with the process, I have repeatedly found that I eventually reach a breakthrough and experience moments of sublime elucidation, revealing important insights and communicating these with clarity. These moments bring a deep satisfaction, and newfound intrinsic motivation where joy re-enters my work.

Oh, but the breakthroughs are hard won! They require a willingness to stay in the mess, to wrestle with complexity, to fight off inner voices that repeatedly tell me I’m not enough. Breakthroughs take time: time to think deeply, to read around the issues and seek broader insight, to allow my thoughts to be evaluated, rather than slapped down with haste in an effort to meet increasingly unrealistic publication targets (Manathunga et al., 2020). It is a systemic *publish or perish* narrative and fixation with output and metrics (Moosa, 2018) that often drive me to feel rushed in this process, rather than affording time for nurturing meaningful work.

**Fig. 4:**
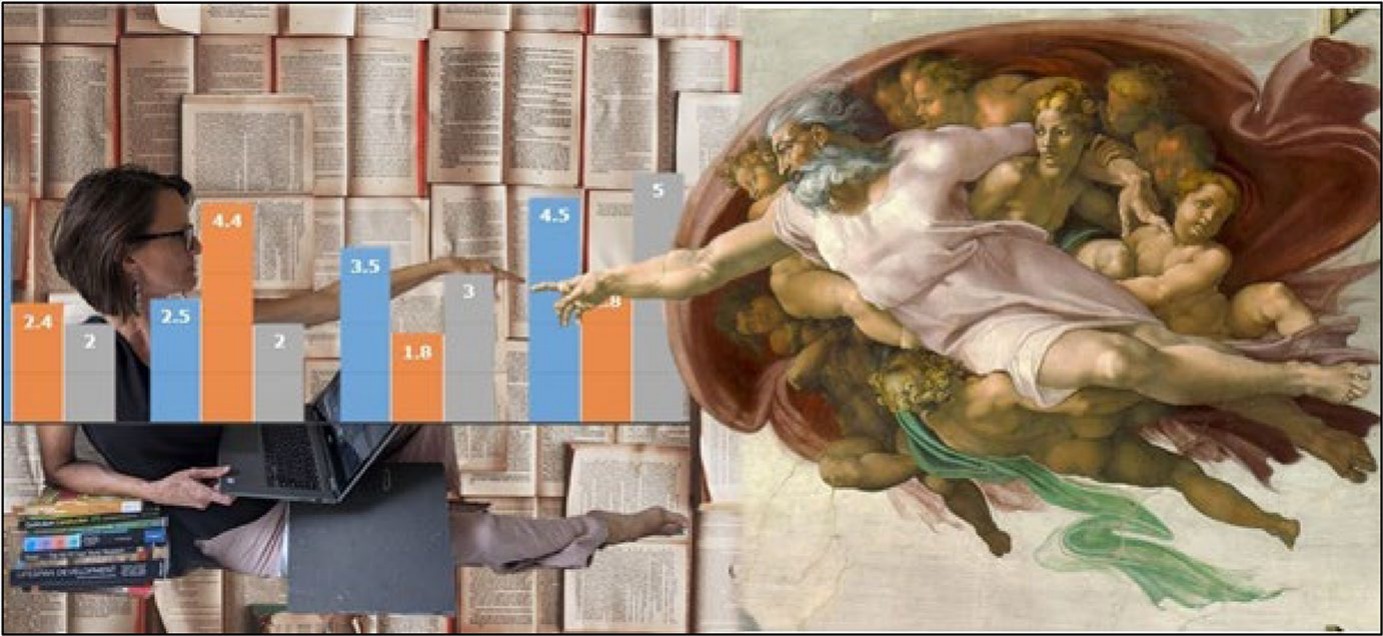
Divine perspiration

My digital collage, Fig. 4, was created to express my feelings around this complexity, and the promise of breakthrough and joy if I persevere. But as the reflective process continued, a deeper story was revealed, of being subsumed into, personally obscured, or lost within, the “machines of institutional academia” (Riddle, 2017, p. 26), and of requirements around research outputs that value metrics above quality contributions (Manathunga et al., 2020). My research sits within a larger story in which I must squeeze the demands of research and writing into an often-overwhelming academic workload which insufficiently allocates time for the complex tasks of teaching, research and service.

It is sobering to consider the thoughts of Nobel prize-winning academic Peter Higgs (of Higgs boson fame), who asserted that today’s ‘new academic culture’ with its incessant demands to keep churning out publications would never have afforded him the time he required to make his very own 1964 breakthrough regarding subatomic theory (Aitkenhead, 2013). These views help me to make clearer sense of why breakthroughs in my work may feel infrequent, unattainable and hard won. Maybe it’s not just me?

The reflective process has helped me to better understand the divine breakthroughs are possible and still happen, but I recognise more clearly how elusive they can be, and why they are often harder to reach than I initially thought. This allows me to extend compassion to myself in this process.

### Overwhelm and optimism

Coming into academia from the background of a primary school teacher and leader, I often feel overwhelmed when it comes to research. While I am confident in the teaching part of my role, I feel less confident putting my thoughts into words, struggling to complete a PhD in a time of changing identity (Bothello & Roulet, 2018).

One element that supports my well-being is being part of an ECR group, which encourages its members to share ideas, anxieties and work together, as well as receiving support from experienced academics. The group has helped me to see that I am not alone; many people share the same insecurities, and I can receive the support required to help build my skills and confidence in writing. Although many times I feel overwhelmed, this group brings me optimism. As Black et al. (2017) describe, I feel inspired when the work is “joyous, meaningful, collaborative and celebratory work” (2017, p. 143).

**Fig. 5:**
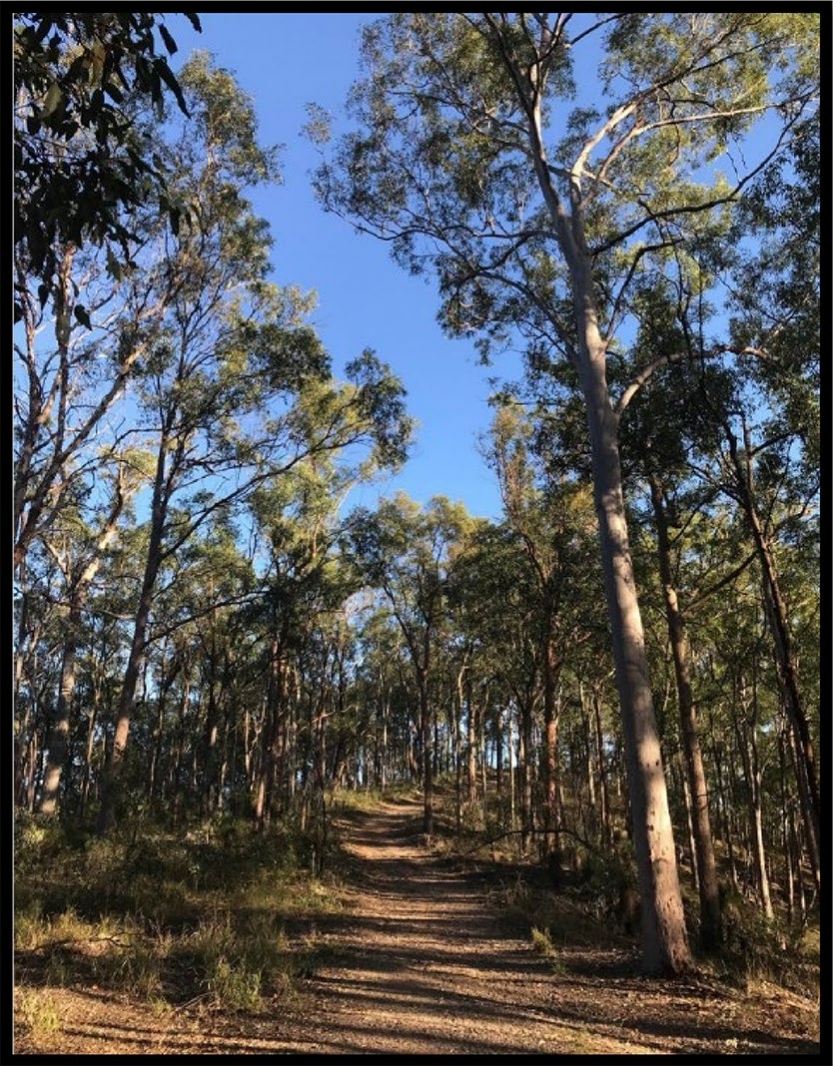
Steep path ahead

In this semester, I have worked on a collaborative piece of writing with a colleague (now published) (Burke & Fanshawe, 2021). The work, alongside my already bursting teaching workload and PhD, can seem unachievable, but I am trying to push towards this goal of completion and publications. Like a large mountain or hill, it seems unattainable from the bottom, however, bit by bit, paragraph by paragraph I am getting closer to the goal (Fig. 5).

I feel the overwhelm comes from my entry to academia later as my second career. I am often anxious about everything pertaining to research and I feel myself saying, “I have no idea what I am doing”. There is a constant feeling of a ‘steep path ahead’ (Fig. 5). This writing group has helped me become more confident, through guiding and lending me a hand, to find strategies that work, providing tips for writing and words of encouragement. I am now in a place of understanding about writing as I have learnt from these guides and angels. I am optimistic that I can push through the overwhelming barriers. I must continue, as I have something to say.

**Fig. 6:**
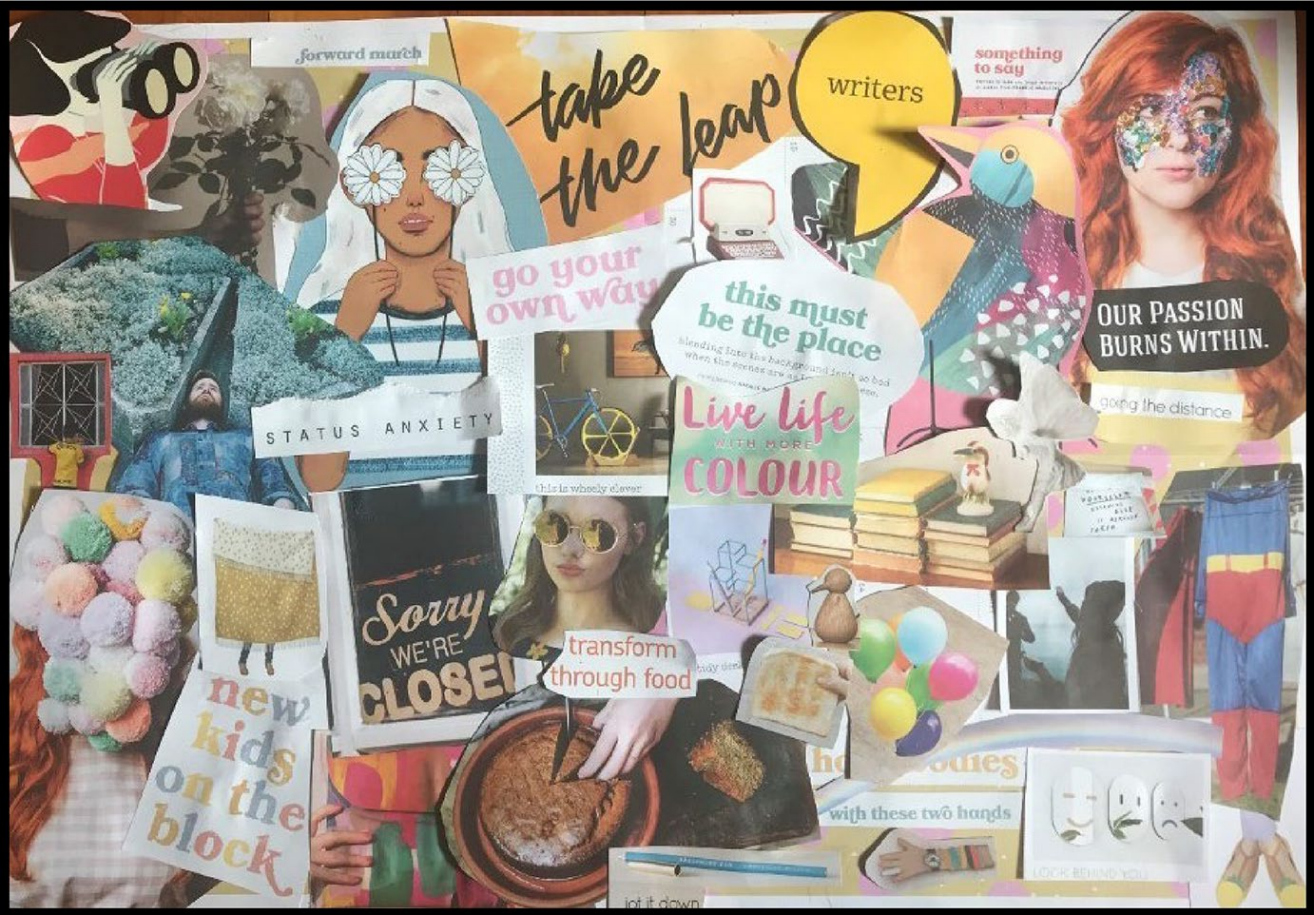
Overwhelm

It is interesting though that as I reflected on my finished recollage (Fig. 6), even my artwork represented more of the overwhelm than the optimism. I am comforted in the words of Condon (2014) who proposes the living experience of feeling overwhelmed has “disconcertedness surfacing with divergent engagements as optimistic anticipation arises” (p. 216). Through the reflective experience, I can see the underlying optimism, which has been shown to be important in ensuring success (Peterson & Chang, 2003) and plays an essential role in mental and physical well-being (Conversano et al., 2010).

### Retrospection

**Fig. 7:**
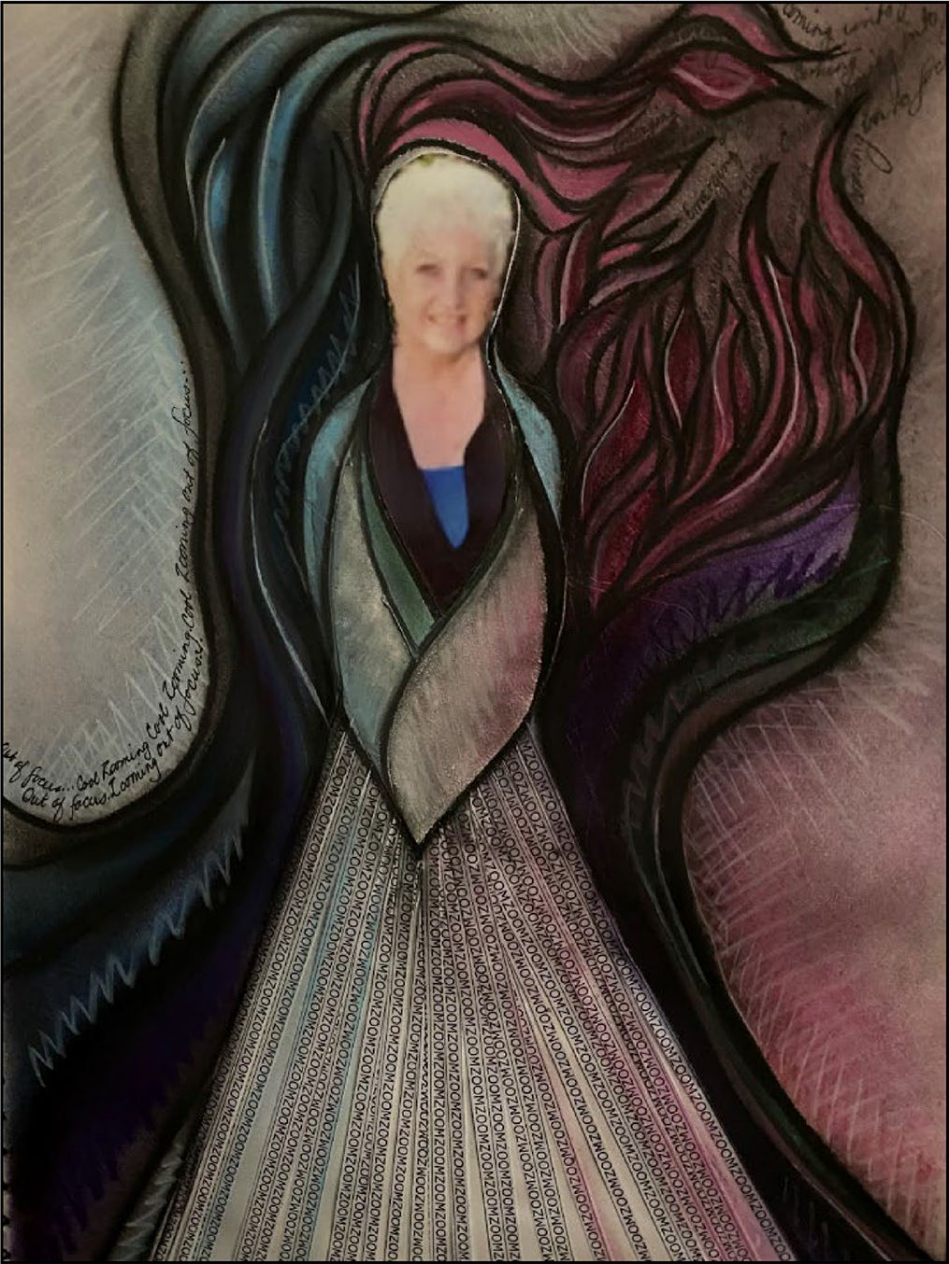
Coming into Focus

Throughout the process of developing artistic endeavours as responses to writing and researching, I found this project’s practical and self-reflective processes' valuable tools for self-development (Creswell, 2012). I have struggled to manage the balance between needing, wanting and being required to accomplish something in the research space. When pondering how I would depict this in retrospect, I considered the stages that led to creating this final image (Fig. 7). Ongoing reflection, in this type of research, for me, is underpinned by two principles:

- development of personal practices which need to be sustained through individual teacher reflection in an ongoing basis, and as Freidhoff (2008) has suggested, based on deep engagement with one’s values, beliefs and assumptions; and
- collaborative practices, occurring in a community setting.

In creating this artwork, I decided that first and foremost I needed to include an image of myself at the centre as I found it was a very personal journey. I also found that the collaborative nature of the process played a crucial role in developing communication that was sincere and the interaction was central to understanding my own personal practices (Creswell, 2012). The collegial aspect of this work was also instrumental in enabling me to move from where I was at the beginning, to where I am now.

**Fig. 8:**
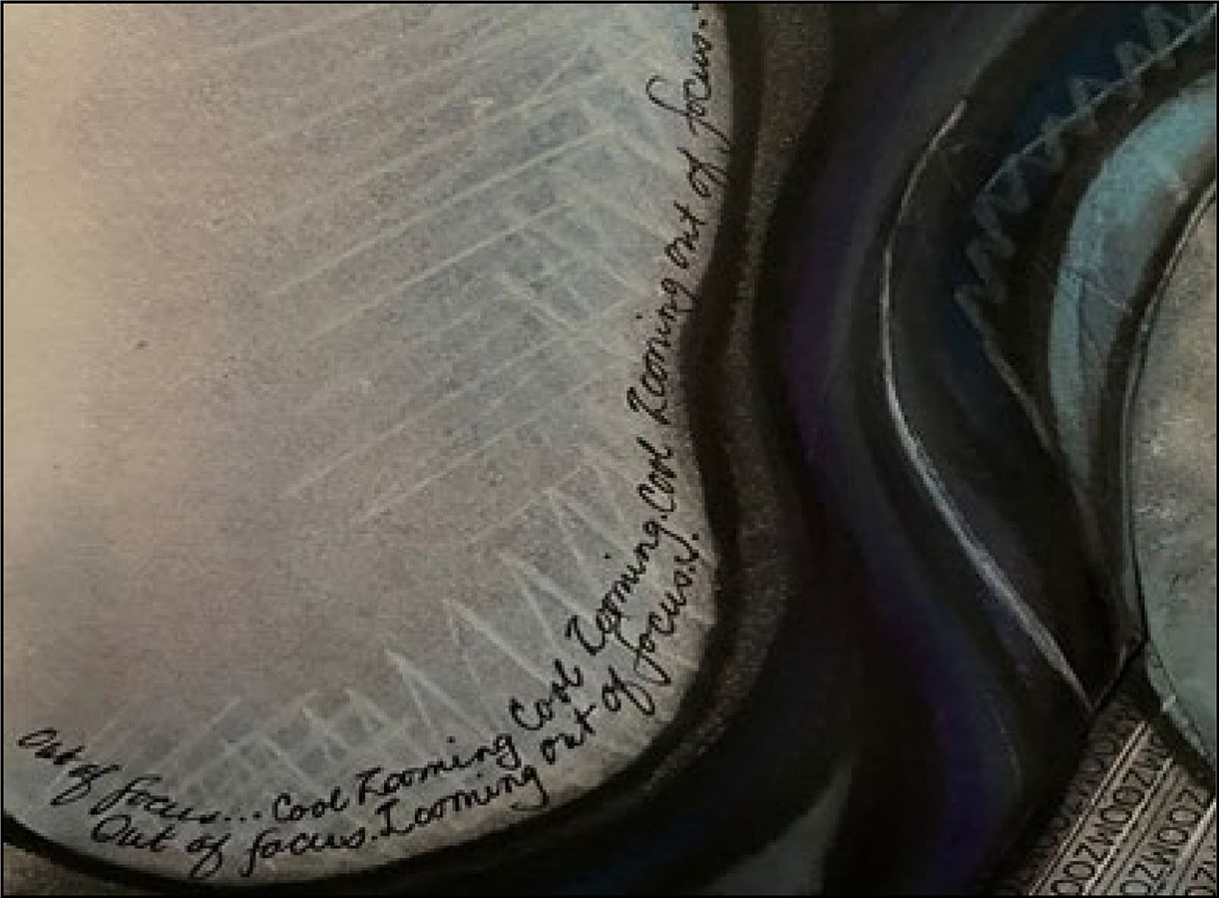
Zooming out of focus excerpt from Coming into Focus

At the beginning I was totally consumed by getting ready to teach for the semester; researching and writing were at the back of my mind and definitely on the back burner. It was like swimming though a blurry treacle mess trying to get my thoughts into focus. And then, there was Zoom. Zoom-zoom as they say in the Mazda ads…. Constant zooming. Zooming for meetings. More meetings because non-zoomers discovered zoom-zooming. More zooming because needy students couldn’t access face-to-face learning. I was totally zooming out of focus (see Fig. 8).

I wanted my image to reflect my part of the journey, from zooming out of focus at the beginning to where I find myself now. Further, as my next artistic endeavours (the collage and Haiku poem) showed, some of my struggles with writing were linked to the difficulty in organising and balancing the professional and personal distractions in my life.

**Fig. 9:**
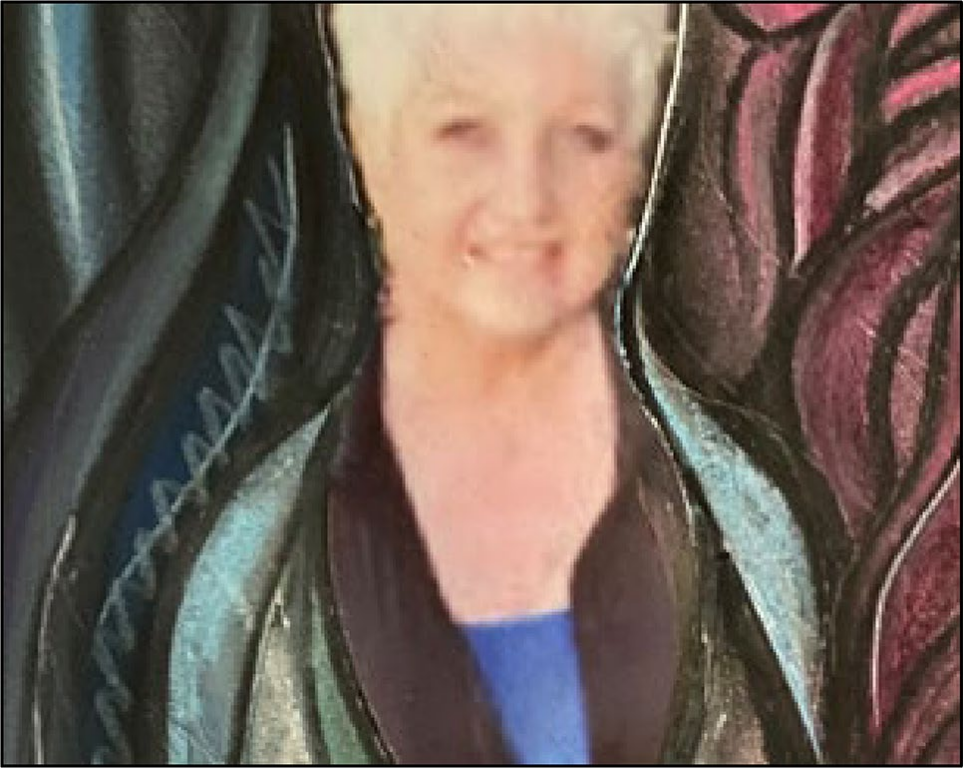
Face out of focus excerpt from Coming into Focus

Like Pagenkopp (2014) found through retrospection, each of the images and pieces created through the arts-based process evolved, merged and were integral to this process. Through the connections created by the images and writing activities, as well as the collegial support and discussions, I now have some clarity about my research and writing (see Fig. 9). Those points along the way where I had to reflect on not just my research or not just my capacity to write, but my whole personal context, pushed and dragged me into a space where I connected myself to the professional research world.

### The inner ‘lion’

**Fig. 10:**
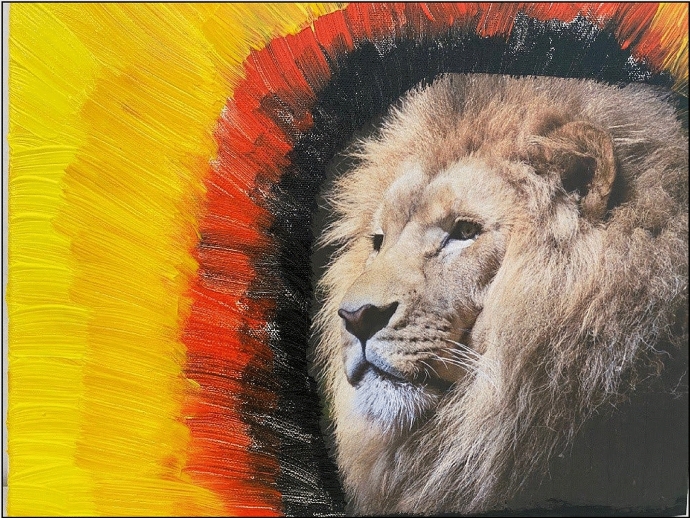
The inner lion

In a professional environment increasingly characterised by the publish or perish dichotomy, I experienced, as an early career academic, significant concern that my perceived value hinged on the writing I produced. Solo authorship exacerbated my fears. Badenhorst et al. (2020) also found sole authoring to be perceived by academics as a far lonelier process evoking greater anxiety. Significant to my writing journey therefore was my progress towards finding my inner lion (see Fig. 10), a source of inner confidence, strength and self-recognition that served to mediate the negative effects of external accountabilities and performative pressures that threatened to undermine my sense of belonging, disrupt the writing process and compromise my ability to ‘keep the love in the work’. Finding the lion within required that I seek out support from trusted colleagues. This step took courage, with Badenhorst et al. (2020) also noting that sole authors are less likely to demonstrate help-seeking behaviours. Courage was repaid, with the voice of this trusted other validating the worthiness of the work, ‘building my own confidence in this piece’ and provoking the lion within me to roar.

My experience clearly demonstrates the importance of a culture of acceptance and support (Badenhorst et al., 2020) within the writing environment, and the availability of trusted others. Badenhorst et al. (2020) call for the academy to take a more humanistic approach to supporting writers with relationship central to the process. This relational ontology seeks not to discourage sole authorship, rather to ensure that all authorship is privy to a “relational holding space” (Badenhorst et al., 2020) so that courage may be fostered.

Courage takes intentional work on the part of the individual academic and those colleagues that surround them. Hayes (2019) likens it to the power of the wolf pack, characterised by the connection between and the courageous contribution of each member of the pack to support each wolf to survive and thrive. According to Hayes (2019), all academics, and in particular ECRs, must find their ‘inner wolf’ in order that fears of professional failure or fraudulence may be managed to create the space for writing to occur. I likened my own courage to that of the lion, enabling me ‘to let the beast of external expectation retreat’ as encouragement and guidance from my lion’s pride, or colleagues, supported me to trust in myself and my work.

### The times they are a-changing

**Fig. 11:**
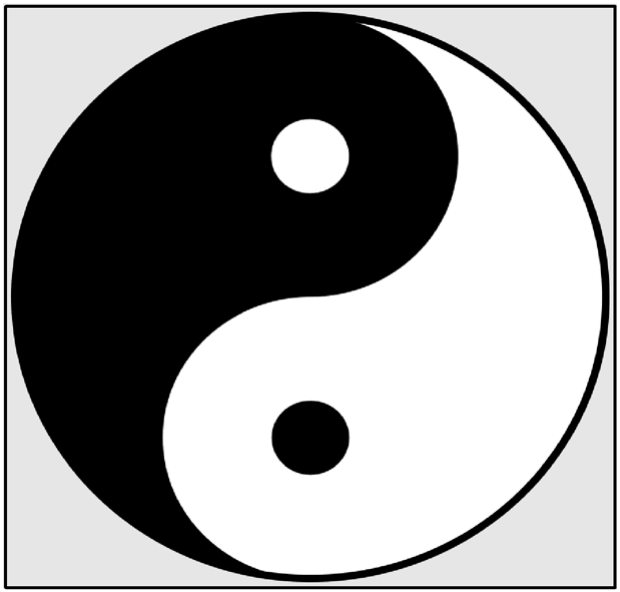
The times they are a-changing

**Fig. 12:**
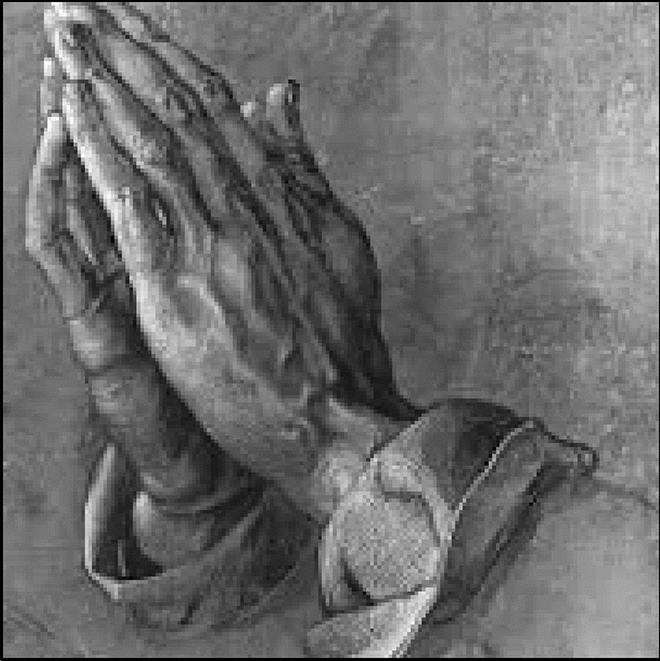
Thank you (NB. Both images are CC)

**Fig. 13:**
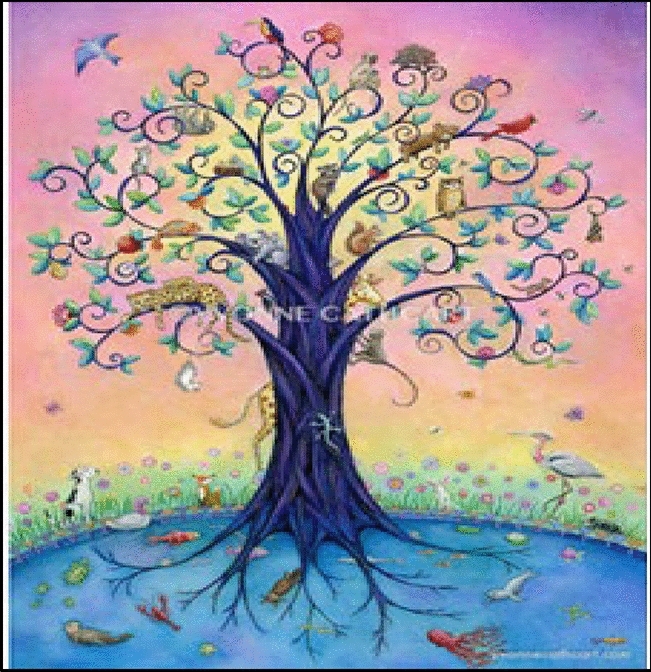
Thank you (NB. Both images are CC)

Balance has always been an important driving force in my process of thinking and writing (Fig. 11). Throughout the process of writing and editing, I endeavour to capture the depth and intensity of the impact of autism on families I have worked with. At the same time, I try to reveal the strength of those marginalised by policy and systems that favour the abled. A seamless emotion that permeates my reflections on writing is *resilience*. The yin and yang image of a circle in its seamless and continuous form and embedded balance between success and failure underpins my spirit of resilience (see Figs. 12 and 13).

Thoughts about my writing are a combination of the voices of people I have listened to, coloured by my own experiences. The families that I work with face a constant struggle with social injustice, marginalisation and discrimination. It is my chosen purpose to listen to the voices of young people and their families and to reveal these to whomever will listen or read my rhetoric. Ultimately, I endeavour to shift archaic community perspectives from a *deficit* to an *abilities* lens for populations with autism. These tentacles of truth and justice extend to education systems of inclusion, disability policy classifications, employers and equality and the broader community in accepting difference and diversity.

Upon reflection, when I look at the images that I have chosen here, they represent things that I hold dearly in my moral ground which link and echo in my practice in writing. Writing for me is a process ever evolving, which is depicted in the Tree of Life image. Sometimes, there is fruit as a reward and other times I have to learn to be happy with the evergreen tree. A difficult lesson to be learnt when rejection comes knocking at the door. It is a journey on a road that is guided by my thoughts, focus and process hence my phrenology head. Of course, the environment, its influence, combined with my inner calm, peace and at times turmoil, is guided only by the will of the universe hence the praying hands giving thanks.

My writing is a process of rhyme and ritual. Candles of all sorts, in those that are meditative (calm, soothing and peaceful) to those that are fragrant and soothe the soul (lavender, sage and sandalwood) adorn my writing space. I quite like music that calms the mind and often have Ravi Shankar Sitar Eclectics or Andres Segovia on my playlist. A constant that inspires the authenticity of my thought process is the eyes of Rumi, Einstein and Mandela strategically positioned above my weathered writing desk.

*From Rumi: I know you’re tired but come, this is the way*.

*From Einstein: Everything should be made as simple as possible, but not simpler*

*From Mandela: It always seems impossible until it's done*

## Discussion and concluding remarks

Working as academics is clearly complex and challenging. Evidence in each of our arts-based reflections shows how we were constantly grappling with the pressures placed upon us to publish in high-quality journals; particularly ECRs and those with caring responsibilities. The process of engaging in arts-based reflections became a cathartic means to better understand these pressures and give voice to more intentional and productive ways of navigating such complexities. While the reflections highlight uniqueness of experience, they collectively also help to generate helpful insight in response to our two research questions:

1. What emotions or metaphors arise when reflecting on the writing process and pressure to publish in the academy? and
2. To what extent does an arts-based approach to reflecting on the writing process and pressure to publish assist in the preservation of self?

Collectively, the reflections and engagement in art-based methodologies revealed the following:

- the power of metaphor to express, more intuitively understand, and elucidate experience. The use of metaphors in both our development of the artefacts and our final reflections demonstrated a re-representation of emotions and experience and presented the opportunity to perceive and experience a shift in conceptualisation through representation (Moser, 2020).
- an expanded understanding through arts-based methods. This permitted us to transform, supplement, enlarge and diversify the tools we used (Barone & Eisner, 2011), notably revealing a consistent and overt desire to experience joy and purpose through our work. This was most potently achieved when we were able to personally invest in what we considered to be meaningful research with impact (notably, impact upon people for positive change, rather than metrics that measured citation scores and journal rankings). The way that self and research were so closely woven together was evident in many reflections. However, what also echoed through the reflections was the way that tokenism, insincere or ‘rushed’ work in response to a performance pressure was the main contributing factor to a loss of the joy, love, hope and optimism that was central to our focus as fruitful academics.

These insights contribute to a chorus of literature that highlights the negative impacts of neoliberal agendas in higher education, obsessions with measurable output and impacts, a publish or perish narrative in higher education, and the impacts upon academic well-being of these (Shin & Jung, 2014; Weisshaar, 2017). Beyond this, the reflections and processes undertaken in this project offer a potential ‘way forward’. The arts-based process of reflection in a supportive group where vulnerability is welcomed permitted each of us to move beyond the privileging of narrowly specified outputs, allowing meaning to be represented in multiple ways, and in itself became an act of self-care and a form of positive resistance that disrupted and extended self-awareness. This became the platform for more meaningful decision-making regarding what to internalise, what to privilege in our work and where to place our energies in the future work.

Metaphors of different natural environments—rough seas, tall trees, winding bush paths and hopeful gardens—revealed the spaces in which we experienced different emotions and were able to satisfy intrinsic needs to transform and manipulate our context (Moser, 2018). How do we find the right key to unlock our potential and achieve a feeling of personal success and accomplishment? Our data show that staying focussed on our passions, knowing what counts to us as well as supporting each other is what will sustain and assist in the process of self-care.
